# Supplementary material for: Psychological morbidity among children with transfusion dependent β-thalassaemia and their parents in Sri Lanka
Source: PLoS One. 2020 Feb 11;15(2):e0228733. doi: 10.1371/journal.pone.0228733 (PMC7012414; doi:10.1371/journal.pone.0228733)
Supplement: Attachment — Submitted filename: Response to reviewers_SM.docx [file pone.0228733.s001.docx]

**Response to Reviewers’ Comments**

**Manuscript title:** Psychological morbidity among children with transfusion dependent beta-thalassaemia and their parents in Sri Lanka

**Manuscript ID:** PONE-D-19-31863

Academic Editor Comments

The reviewer’s comments are positive, however the following two minor revisions will, I feel, enhance the value of this paper. I would recommend the authors to make these revisions and submit.

- The relationship between maternal depressive symptoms and clinical characteristics of the children (e.g. frequency of blood transfusions) would be interesting to analyze.
- It would be interesting to include a short paragraph regarding the limitations of the study (if any) in the discussion section.

**Author Response:** We have now addressed above two comments raised by the reviewers in the revised manuscript. (Please see new table 6, results section and discussion)

Reviewer #1 Comments

1. The use of a case-control study design was appropriate to meet the objectives.

**Author Response:** No revisions were required.

2. The relationship between maternal depressive symptoms and clinical characteristics of the children (e.g.frequency of blood transfusions) would be interesting to analyze.

**Author Response:** We have now included analysis of maternal depressive symptoms and clinical characteristics of children in the revised manuscript (new table 6).

3. The findings are well presented and has important implications for the discipline.

**Author Response:** We thank reviewer for this positive comment.

4. It would be interesting to include a short paragraph regarding the limitations of the study (if any) in the discussion section.

**Author Response:** We have now included a paragraph on limitations of the study in the discussion.

Reviewer #2 Comments

1. Psychological morbidity among children with transfusion dependent beta-thalassaemia and their parents in Sri Lanka is an interesting well written article focus on important area of health, the title and abstracts were consistent with the presented problem, the background is clear and easy to understand even for those not in the field the result are credible the conclusion is logic and valid.

**Author Response:** We thank reviewer for this positive comment.
